# Photodynamic Therapy for Eye, Ear, Laryngeal Area, and Nasal and Oral Cavity Diseases: A Review

**DOI:** 10.3390/cancers16030645

**Published:** 2024-02-02

**Authors:** Wojciech Domka, Dorota Bartusik-Aebisher, Wiktoria Mytych, Angelika Myśliwiec, Klaudia Dynarowicz, Grzegorz Cieślar, Aleksandra Kawczyk-Krupka, David Aebisher

**Affiliations:** 1 Department of Otolaryngology, Medical College of The University of Rzeszów, 35-959 Rzeszów, Poland; w.domka@gazeta.pl; 2 Department of Biochemistry and General Chemistry, Medical College of the University of Rzeszów, 35-959 Rzeszów, Poland; dbartusikaebisher@ur.edu.pl; 3 Students English Division Science Club, Medical College of The University of Rzeszów, 35-959 Rzeszów, Poland; wiktoriamytych@gmail.com; 4 Center for Innovative Research in Medical and Natural Sciences, Medical College of The University of Rzeszów, 35-310 Rzeszów, Poland; amysliwiec@ur.edu.pl (A.M.); kdynarowicz@ur.edu.pl (K.D.); 5 Department of Internal Diseases, Angiology and Physical Medicine, Centre for Laser Diagnostics and Therapy, Medical University of Silesia, Batorego 15, 41-902 Bytom, Poland; cieslar1@tlen.pl; 6 Department of Photomedicine and Physical Chemistry, Medical College of the University of Rzeszów, 35-959 Rzeszów, Poland

**Keywords:** photodynamic therapy (PDT), head area, carcinoma

## Abstract

**Simple Summary:**

Photodynamic therapy (PDT) has emerged as a versatile treatment option for head and neck disorders. The purpose of our review was to provide an overview of the use of PDT therapy in a variety of diseases. The use of PDT therapy has recently emerged as an innovative and effective method of cancer treatment. However, more and more studies are showing that it is also promising for treating a variety of non-cancerous lesions, providing a minimally invasive alternative for certain conditions. The same following principles apply: the application of a photosensitizer, exposure to light, and then the destruction of abnormal cells. In our work, we highlighted the importance of photodynamic therapy and its valuable treatment as an option for a variety of diseases of the head and neck area, not just cancer. With this, we want to encourage researchers to more widely use photodynamic therapy in head and neck disorders.

**Abstract:**

Photodynamic therapy (PDT) has emerged as a promising modality for the treatment of various diseases. This non-invasive approach utilizes photosensitizing agents and light to selectively target and destroy abnormal cells, providing a valuable alternative to traditional treatments. Research studies have explored the application of PDT in different areas of the head. Research is focusing on a growing number of new developments and treatments for cancer. One of these methods is PDT. Photodynamic therapy is now a revolutionary, progressive method of cancer therapy. A very important feature of PDT is that cells cannot become immune to singlet oxygen. With this therapy, patients can avoid lengthy and costly surgeries. PDT therapy is referred to as a safe and highly selective therapy. These studies collectively highlight the potential of PDT as a valuable therapeutic option in treating the head area. As research in this field progresses, PDT may become increasingly integrated into the clinical management of these conditions, offering a balance between effectiveness and minimal invasiveness.

## 1. Introduction

### 1.1. Head and Neck Cancers

Head and neck cancers account for around 4–5% of all cancers worldwide [1]. In 2020, there were an estimated 890,000 new cases of lip and oral cavity cancer and 650,000 new cases of laryngeal cancer globally. The mortality rate for head and neck cancers is significant, and these cancers are responsible for a substantial number of cancer-related deaths worldwide. In 2020, there were an estimated 450,000 deaths from lip and oral cavity cancer and 330,000 deaths from laryngeal cancer [2,3].

### 1.2. Main Areas

**Figure 1:**
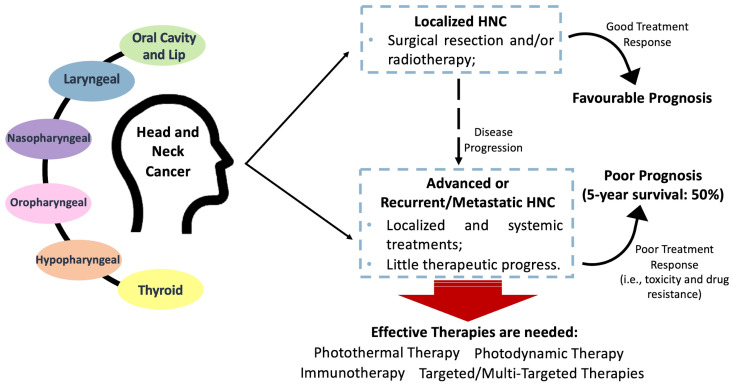
Head and neck cancer treatment prognosis.

Cancers of the head and neck refer to a group of cancers that occur in the oral cavity, throat (pharynx), voice box (larynx), paranasal sinuses, nasal cavity, and salivary glands. These cancers can affect various structures in the head and neck region. Common risk factors for head and neck cancers include tobacco use (smoking or chewing), excessive alcohol consumption, human papillomavirus (HPV) infection, and exposure to certain workplace carcinogens (like asbestos). Symptoms may vary depending on the location and stage of the cancer but can include persistent sore throat, difficulty swallowing, changes in voice, a lump or sore that does not heal, and unexplained weight loss. Early detection is crucial for successful treatment [4,5]. Regular dental check-ups and health screenings can aid in the early detection of head and neck cancers [6,7]. Figure 1 shows head and neck cancer treatment prognosis.

### 1.3. Photodynamic Therapy

PDT has garnered significant attention and application in otolaryngology, showcasing its versatility and effectiveness in addressing a spectrum of disorders in the nose, mouth, throat, and related structures [8]. The upper respiratory system, comprising intricate anatomical components such as the nasal cavity, oral cavity, larynx, and adjacent tissues, presents a unique set of challenges in the realm of medical treatment. Traditional approaches, while effective, may entail invasive procedures and potential damage to healthy tissues. PDT, however, emerges as a promising alternative, offering a delicate balance between precision and therapeutic impact [9,10].

The fundamental principle of PDT involves the administration of a photosensitizing agent, often activated by light of a specific wavelength. This activation induces a localized photochemical reaction, generating reactive oxygen species that selectively target and eliminate abnormal or cancerous cells. The remarkable advantage of PDT lies in its ability to spare adjacent healthy tissues, preserving vital structures and minimizing collateral damage [11]. Research studies have delved into the application of PDT in diverse upper respiratory conditions. From treating laryngeal tumors with a focus on tissue preservation to enhancing vocal fold wound healing and managing complications of laryngotracheal stenosis, PDT demonstrates a wide range of therapeutic potentials. Additionally, its role in addressing premalignant lesions in the oral cavity and juvenile-onset laryngeal papillomatosis highlights the versatility of this approach. Studies investigating PDT in the treatment of nasal cavity and paranasal sinus pathologies have shown encouraging results [12,13]. Photosensitizers, such as 5-aminolevulinic acid (ALA) and hematoporphyrin derivatives, have been employed to selectively target neoplastic lesions. The advantage of PDT lies in its ability to precisely target abnormal tissue, minimizing damage to surrounding healthy structures. PDT has demonstrated efficacy in the management of conditions like sinonasal inverted papillomas and early-stage malignancies [14]. In the larynx, PDT has been explored for its potential in treating various disorders, including laryngeal cancers and benign lesions. The use of photosensitizers like protoporphyrin IX (PPIX) induced by ALA has shown promising outcomes. One key advantage of PDT in laryngeal applications is its capacity to preserve laryngeal function and structure. Studies have reported positive effects on vocal fold wound healing, reduction in scar formation, and overall improvement in voice outcomes [15]. Optimal outcomes in PDT depend on various parameters, including the choice of photosensitizer, light source characteristics, and treatment timing. The concentration of photosensitizers, such as ALA or hematoporphyrin derivatives, needs careful consideration to achieve the desired therapeutic effects while minimizing damage to healthy tissues. Additionally, the selection of an appropriate light source in terms of wavelength and energy delivery plays a crucial role in the success of PDT interventions. While PDT holds significant promise in the upper respiratory system, challenges such as the depth of light penetration and the need for repeated treatments in certain cases remain. Future research aims to address these challenges and explore novel photosensitizers and light sources to enhance the efficacy of PDT. Furthermore, efforts are underway to expand the indications for PDT in the upper respiratory system, including its application in inflammatory and infectious conditions [16]. This compilation of research underscores PDT’s significance in otolaryngology, showcasing its potential as an organ- and function-preserving treatment modality. As the medical community continues to explore and refine the applications of PDT, it becomes increasingly evident that this technique holds promise as a valuable tool in the nuanced landscape of upper respiratory system disorders. The following sections provide a closer look at specific applications of PDT in various regions of the upper respiratory system, shedding light on its efficacy and potential impact on patient outcomes. Diagnosis frequently begins with the first symptoms of cancer that the patient notices. Symptoms are related to the function this organ performs in the body. For example, the larynx is primarily responsible for breathing, speaking, and protecting the airway during swallowing. Patients may report shortness of breath, a change in the sound of their voice, hoarseness, difficulty swallowing, or coughing [17,18]. It should be mentioned that these symptoms depend on the location of the tumor.

### 1.4. Mechanism of Photodynamic Therapy

**Figure 2:**
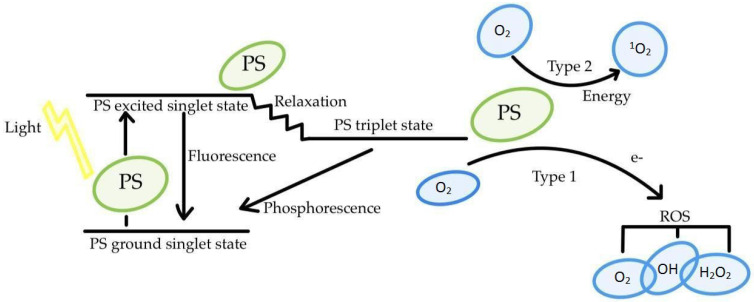
Mechanism of PDT.

PDT is a method that uses a light-sensitive dye and light of the appropriate wavelength in the presence of molecular oxygen [19] (Figure 2). Recently, a number of studies have aimed to test the effects of PDT on the treatment of laryngeal diseases, including premalignant lesions and malignancy itself [20]. Photodynamic therapy, due to its possession of three components, is referred to as a photochemotherapeutic method [21]. It is also widely used in dermatology, dentistry, and ophthalmology and exhibits bactericidal activity [22]. Using the desired physicochemical reactions ultimately leads to the apoptosis of tumor tissue [23]. PDT therapy involves the use of a suitable photosensitizer, which accumulates in the decedent—cancerous tissue [24]. Cellular hypoxia is one of the main problems in the transport of drugs, leading to the inactivation of their action [25]. Scientists have shown that hypoxic tumor cells are resistant to PDT. It has been noted that more than half of advanced cancer tumors have areas of hypoxia and/or anoxia [26]. These may be due to the physiological properties of the tumor cells or may be the result of oxygen depletion during PDT therapy [27,28,29]. Therefore, a necessary ingredient during PDT therapy is an adequate amount of dissolved molecular oxygen in the tissues during irradiation [30]. Molecular oxygen is the substrate for the photochemical reactions that accompany PDT, leading to the formation of singlet oxygen and other reactive oxygen species [31] (Figure 2). The basic state for molecular oxygen is the triplet state; it has two unpaired electrons in p orbitals and a total electron spin of S = 1. The singlet state is characterized by two paired electrons and a resultant electron spin of S = 0 [32].

Due to the presence of oxygen, the excited molecule can react directly with the substrate by transferring a proton or electron, thus forming radicals or radical ions, which interact with oxygen to form oxidized products—a type I reaction [33]. Directly transferred photosensitizer energy to oxygen forms singlet oxygen—a type II reaction [34,35]. Singlet oxygen is the most harmful compound generated during PDT therapy [36]. In an environment rich in molecular oxygen, a type II reaction takes place, so singlet oxygen is generated. Singlet oxygen is a metastable form and has high reactivity, leading to cell destruction. Singlet oxygen is more desirable due to its longer life span compared to other radicals. In the cellular environment, singlet oxygen lives for about 0.5 μs, allowing it to diffuse over distances of 0.1–0.2 μm [37,38,39]. In the cell, singlet oxygen can interact with other molecules by transferring its excitation energy (return to triplet state) or participating in a chemical reaction [40]. The irradiation of tumor tissues with the optimal wavelength of light and oxidation with singlet oxygen or radicals leads to the necrosis of this tissue [41]. PDT therapy uses non-ionizing light in absorption. The wavelength of the light must be matched to that of the photosensitizer [42]. The 600–1300 nm spectral region, where there is minimal light absorption, is often referred to as the “optical window” of the tissue [43,44]. At the short wavelength end, the window is limited by the absorption of oxy/deoxyhemoglobin. At the long wavelength end of the window, light penetration is limited by the immersion parcels of water. The optimal light diapason for PDT remedy is a wavelength of 600-800 nm. The use of longer wavelengths is ineffective in converting oxygen motes from the ground state to the agitated state [45]. The interactions between light and towels include reflection, refraction, scattering, and immersion. Reflection and refraction are described by Fresnel’s law and Snell’s law. Scattering leads to the scattering of light and the reduction in its intensity [46]. Depending on the size of the patches in the towel relative to the wavelength of the incident light, Rayleigh or Mie scattering formalism can be used. The main largely absorbing motes in apkins include water, oxyhemoglobin, deoxyhemoglobin, melanin, and cytochromes. When light is absorbed, the absorbed radiation is substantially converted to heat by the towel’s absorbing chromophore. The scattering and absorption of light in tissue are determined by the scattering and absorption coefficient [47]. Both scattering and absorption remove energy from a beam of light passing through a medium, leading to the attenuation of the beam. There is a large range of distance-dependent scattering and absorption coefficients in different tissues, which affects the penetration and distribution of light in the volume. The depth of optical penetration in tissue is wavelength dependent due to the cellular content and chromophores in the tissue having wavelength-dependent scattering and absorption properties [48,49,50,51,52,53]. A very important feature of PDT is that cells cannot become immune to singlet oxygen [54]. During PDT therapy, there is a level of singlet oxygen concentration above the physiological range, caused by oxidative stress [55]. Entering cells in large quantities will damage the amino acid residues of proteins, components of nucleic acids, and lipids to a very high degree [56,57]. Apoptotic pathways are activated, or proteins are damaged leading to cell impairment [58]. The induction of sufficient triplet oxygen occurs by supplying sufficient energy [59]. An endogenous photosensitizer excited by the light radiation of the appropriate wavelength transfers its excitation energy to oxygen by which the photosensitizer returns to the basal state and oxygen is converted to singlet oxygen [60,61]. The effect of the therapy is also affected by the specific structure of cancer tumors [62]. The concentration of the drug decreases in cells far from the lumen of the vessel. Therefore, it often does not reach their central part at all, making the therapy ineffective. The use of drug carriers makes it possible to control the transport of substances to their destination and their selective apoptosis by activating photobiochemical processes. By coating the molecules of substances with a photosensitive coating, we can control the combination of molecules and the precise transport of drugs into the body. This means that we can control them once they enter the body by responding to light emission. With this therapy, patients can avoid lengthy and expensive surgeries. PDT therapy is referred to as a safe and highly selective therapy [63,64,65]. Currently, photosensitizers are still being sought that will be more effective, safer, and less invasive to humans [66,67,68,69,70]. UV-Vis spectroscopy is one of the methods by which photosensitizers are analyzed [71]. Important features that a photosensitizer should have include the following: low toxicity before irradiation (no cytotoxicity), high efficiency in generating reactive oxygen species after irradiation, chemical stability, chemical purity, high quantum efficiency of triplet state formation, no aggregation phenomenon, no side effects in the body, rapid removal from the body, no effect on healthy tissue, absorption in the range of 600–800 nm, selective accumulation in tumor tissue, and water solubility [72].

### 1.5. Pros and Cons

Photodynamic therapy (PDT) has emerged as a promising and versatile treatment modality in the realm of healthcare, particularly in addressing various conditions in the head area. The unique advantage of PDT lies in its non-invasiveness, precision, and adaptability, making it particularly well suited for applications in the complex anatomy of the head. The head is a complex and vital region of the human body, encompassing various crucial structures and sensory organs. This area plays a central role in communication, perception, and basic physiological functions. The head area is not only anatomically intricate but also a hub for various sensory and motor functions. It is a convergence point for the nervous, respiratory, digestive, and auditory systems, making it a critical region for overall health and well-being.

### 1.6. Photodynamic, Radiological, and Surgical Therapies

Photodynamic therapy (PDT), radiological therapy, and surgical therapy represent distinct modalities in the management of head and neck diseases, each with its unique characteristics, advantages, and limitations. A comprehensive comparison of these therapeutic approaches provides insights into their relative merits and suitability for different clinical scenarios (Table 1).

The choice between photodynamic therapy, radiological therapy, and surgical therapy in head and neck diseases depends on factors such as the nature of the disease, tumor characteristics, patient preferences, and available resources. Each modality has its place in the multidisciplinary management of these conditions, and the selection should be tailored to the individual patient’s needs and the specific clinical scenario.

## 2. Methodology

**Figure 3:**
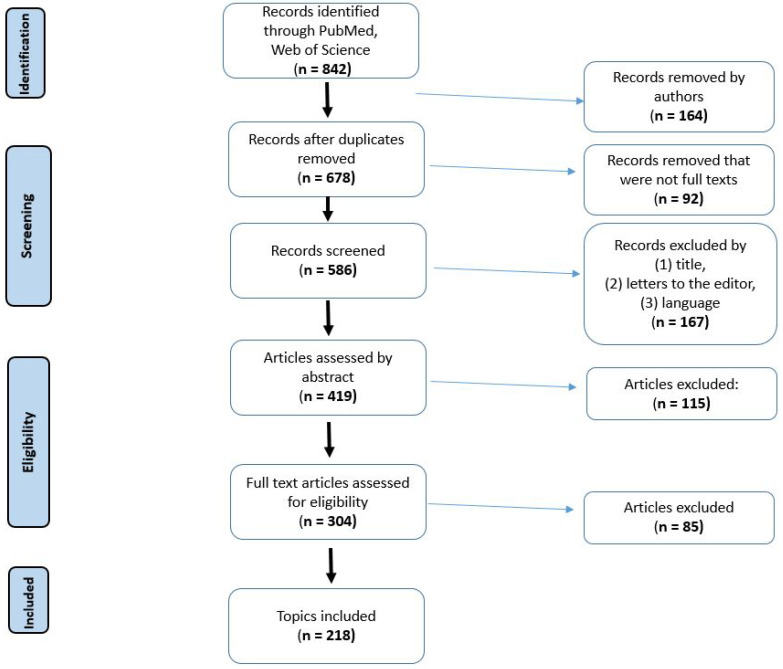
PRISMA flow diagram of included studies.

A search focused on the effectiveness of PDT in laryngeal cancer was conducted on Pubmed and Web of Science from 1997 (only one from 1969). This review was conducted based on the Preferred Reporting Items for Systematic Reviews and Meta-Analyses (PRISMA) guidelines. The search term included the following phrases: “photodynamic therapy for eyes diseases”, “photodynamic therapy for ears diseases”, “photodynamic therapy for nasal cavity”, “Laryngeal cancer photodynamic therapy” and “photodynamic therapy for oral cavity”. This review included both in vivo clinical studies and in vitro preclinical studies. Duplicate records were removed. Figure 3 shows the PRISMA diagram of the included studies.

## 3. A Review of the Literature

### 3.1. PDT Therapy in Eyes

These studies collectively highlight the diverse applications and effectiveness of photodynamic therapy in treating various eye conditions, ranging from chronic central serous chorioretinopathy and polypoidal choroidal vasculopathy to choroidal neovascularization and retinoblastoma. PDT has shown promising outcomes in terms of anatomical resolution, visual acuity improvement, and safety across different eye disorders.

#### 3.1.1. Chronic Central Serous Chorioretinopathy (cCSC)

- A randomized controlled trial found that half-dose PDT was more effective than a high-density subthreshold micropulse laser (HSML) in resolving subretinal fluid (SRF) and improving visual outcomes in cCSC patients [91].
- Crossover to half-dose PDT after unsuccessful HSML treatment showed improved anatomical and functional outcomes in cCSC patients [92].
- A multicenter follow-up study revealed that cCSC patients treated with half-dose PDT were less likely to experience SRF recurrences compared to HSML at 20 months after treatment [93].
- Reduced doses of verteporfin PDT were found to significantly impact choroidal blood flow in chronic CSC [94].
- A prospective series indicated that primary PDT is a safe and efficient treatment for small pigmented posterior pole choroidal melanoma, offering short-term tumor control and preserving vision [95].
- A follow-up study in cCSC patients who achieved a complete resolution of SRF with either half-dose PDT or HSML showed that those treated with half-dose PDT were less likely to experience SRF recurrence [93].
- Evaluations of one-third dose verteporfin PDT in chronic CSC demonstrated improvements in choroidal thickness, central choroidal capillary layer thickness, and best-corrected visual acuity (BCVA) [96].
- A study comparing different doses of verteporfin PDT in chronic CSC indicated a significant impact on choroidal blood flow, with variations depending on the verteporfin dose [94].
- Topographical changes in choroidal thickness after PDT for CSC revealed a significant decrease in choroidal thickness up to 3 mm from the fovea [97].
- Another study on chronic CSC patients treated with half-dose PDT showed a significant decrease in subfoveal choroidal thickness after treatment [98].

#### 3.1.2. Polypoidal Choroidal Vasculopathy (PCV)

- The EVEREST II trial suggested that combination therapy with ranibizumab and verteporfin PDT is efficacious and safe for treating PCV, achieving better BCVA gain, increased odds of complete polypoidal lesion regression, and fewer treatment episodes compared to ranibizumab monotherapy [99].
- A randomized clinical trial involving Asian participants with symptomatic macular PCV demonstrated that combination therapy with ranibizumab and verteporfin PDT was not only non-inferior but also superior to monotherapy in terms of BCVA improvement and complete polyp regression [100].
- In a case study, a rare side effect of PDT for circumscribed choroidal hemangioma was observed, leading to the development of polypoidal choroidal vasculopathy (PCV) [101].
- Combined treatment with ranibizumab and verteporfin PDT was found more effective than PDT alone in patients with predominantly classic choroidal neovascularization (CNV) secondary to neovascular age-related macular degeneration [102].
- A study exploring the outcomes of combined intravitreal triamcinolone and PDT with verteporfin for subfoveal CNV caused by AMD suggested better outcomes compared to PDT monotherapy [103].

#### 3.1.3. Other Conditions and Studies

- A study explored the potential use of PDT for treating retinoblastoma, indicating a higher phototoxic effect in cancer cells compared to normal cells [104].
- Combining trabeculectomy with PDT was investigated as a new approach to modulate postoperative wound healing in glaucoma patients [105].
- An extrafoveal PDT occlusion of feeder vessels in patients with subfoveal CNV due to AMD demonstrated potential improvements in central vision without causing subfoveal retinal damage [106].

### 3.2. PDT Therapy in Ears

These studies collectively highlight the versatility of PDT across different conditions, showcasing its effectiveness in achieving regression, providing non-surgical alternatives, and presenting promising outcomes for challenging cases.

#### 3.2.1. Posterior Choroidal Amelanotic Melanomas

Primary PDT demonstrated high efficacy, with 88% experiencing complete regression. Notable recurrence rates (36%) emphasize the need for close follow-up. PDT provided a vision-preserving treatment option for these melanomas [107].

#### 3.2.2. Basal Cell Carcinoma (BCC) in the External Auditory Canal (EAC)

In the successful treatment of BCC in the EAC with PDT using a 5-ALA solution, PDT proved effective as a non-surgical option for BCC, particularly for patients unwilling or unable to undergo surgery [108].

#### 3.2.3. Vessel Sclerosis in Telangiectasia

PDT was significantly more effective in inducing target vessel sclerosis compared to glucose injection and controls. PDT led to reduced vessel detection and temperature. While not significantly different, one case of necrosis was observed in the PDT group [109].

#### 3.2.4. Angiolymphoid Hyperplasia with Eosinophilia (ALHE)

The combination of electrocoagulation and PDT showed promise for ALHE, achieving complete regression in two patients. The combination treatment demonstrated favorable outcomes, suggesting potential efficacy, particularly in challenging anatomical locations [110].

### 3.3. PDT Therapy in Nasal Cavity

These studies collectively showcase the diverse applications and promising outcomes of PDT across various medical conditions, ranging from infections to cancer treatment, highlighting its efficacy and potential as a safe alternative or supplementary therapy.

#### 3.3.1. *Staphylococcus aureus* (S.A) Inhibition

PDT using curcumin and methylene blue (MB) showed notable antimicrobial efficacy against S.A, especially with extended laser irradiation. However, chlorhexidine (CHX) mouthwash was superior in achieving the minimum colony count [111].

#### 3.3.2. Nasopharyngeal Cancer Treatment

Photofrin PDT showed better local response and nasal cavity obstruction remission rates compared to chemotherapy in recurrent nasopharyngeal cancer patients. It improved patients’ quality of life, suggesting PDT as a safe and effective treatment for advanced cases [112].

#### 3.3.3. Recurrent Tumors in Paranasal Sinuses

PDT as an adjuvant therapy to salvage surgery for recurrent tumors in paranasal sinuses was safe and achieved complete responses in some cases. PDT emerged as a feasible supplementary treatment, particularly when complete resection was challenging [113].

#### 3.3.4. Intranasal PDT for SARS-CoV-2 Carriers

Intranasal PDT significantly reduced SARS-CoV-2 infectivity, slowed immune response decline, and was safe for mildly symptomatic COVID-19 patients. It provided a potential intervention to reduce the infectivity period [114].

#### 3.3.5. Photodynamic Therapy for Rhinosinusitis

Curcumin-based PDT demonstrated efficacy against nasal bacteria, suggesting PDT as a relevant option for controlling rhinosinusitis [16].

#### 3.3.6. PDT against Antibiotic-Resistant Bacteria

ALA-mediated PDT effectively reduced bacterial colony formation in antibiotic-resistant strains of Staphylococcus aureus and Staphylococcus epidermidis obtained from chronic rhinosinusitis patients [115].

#### 3.3.7. Light Dosimetry in Sinonasal PDT

Sinonasal PDT showed significant temporal and spatial variations in fluence rate. This study emphasizes the need for improvement in in vivo light dosimetry for PDT [116].

#### 3.3.8. Needleless Jet Injection for BCC PDT

Needleless jet injection enhanced the topical delivery of aminolevulinic acid (ALA) for PDT in treating nodular basal cell carcinoma on the nose. The procedure demonstrated good cosmesis and promising clinical outcomes [117].

#### 3.3.9. HpD-PDT for Recurrent Nasopharyngeal Cancers

PDT using hematoporphyrin derivative (HpD) effectively controlled recurrent or residual nasopharyngeal cancers, achieving long-term tumor control in localized lesions and inducing responses in advanced cases [118].

### 3.4. PDT Therapy in the Laryngeal Area

Laryngeal carcinomas represent one of the most important problems when it comes to diseases of the head and neck region. It is estimated that among new cancers diagnosed annually worldwide, laryngeal malignancy accounts for about 3% [119]. The five-year survival rate currently ranges from 53 to 62%, according to the ACS, showing that treatment methods could be more effective [120].

Zhang C. et al., in their work, examined the effectiveness of PDT therapy on early-stage laryngeal dysplastic cells and papilloma. Cells were harvested from one patient in vitro. PDT testing on primary human vocal fold fibroblasts in vitro was performed using Cell Counting Kit-8, real-time polymerase chain reaction, immunoassay, and Western blotting. The results showed the formation of anti-fibrotic lesions in vocal fold fibroblasts. Photodynamic therapy changed the expression profile of fibroblasts and induced an anti-fibrotic effect [121].

Advanced stages of laryngeal carcinoma can even lead to the removal of the entire organ or death of the patient, which is why early diagnosis and comprehensive therapy are so important. Standard treatments include surgery, radiation therapy, and chemotherapy, but as studies have shown, these methods are fraught with many complications and side effects, as well as carcinoma recurrence. As a result, a lot of research is focusing on more and more new solutions and treatments for cancers, including laryngeal malignancy. One of these methods is PDT. The purpose of this study was to evaluate the effects of photodynamic therapy on laryngeal diseases, including laryngeal malignancies and precancerous stages.

Li et al. investigated the effects of PDT in patients with adult-onset laryngeal papillomatosis (ALP). In their study, the authors presented three case reports of patients with ALP recurrence after surgical treatment, microdebridization, and CO_2_ laser therapy, who were treated with a combination of 5-aminolevulinic acid photodynamic therapy (ALA-PDT) with carbon dioxide (CO_2_) laser therapy. The patients were examined 6 and 12 months after the therapy, and all were considered cured, with none of the patients experiencing recurrences. The authors report that ALA-PDT can eliminate abnormal proliferating cells without causing damage to healthy tissue, so there is potential for this treatment method. However, further research is needed on ALA-PDT for the treatment of ALP [122].

Similar conclusions were reached by Liang et al. who performed a study on thirteen patients, including nine pediatric and four adult patients with laryngeal papillomatosis (LP) who were treated with local ALA-PDT after tumor resection (CO_2_ laser resection and/or microdebrider) at the time. According to the authors, follow-ups of the patients 12 months after treatment showed that 84.6% of the cases showed no recurrence, while recurrences occurred in two cases (15.4%). One of the treated pediatric patients developed dyspnea due to laryngeal mucosal edema, while four patients had adhesion of the anterior vocal cord conjunctiva. The authors concluded that ALA-PDT can reduce LP recurrence and improve the cure rate, while further research is needed on the effects and side effects of PDT [123].

Toksoy A. et al. described the effects of in vitro PDT therapy with Norsquaraine 1 using Hep-2 laryngeal epidermoid carcinoma cells. An increased reactivity of oxygen species was observed. In addition, the use of PDT in combination with Norsquaraine 1 yielded a high efficacy of 77–89% cachexia of the tumor cells [124].

Zhang Y. et al. conducted a study of the effect of temperature on laryngeal squamous cell carcinoma cells using delta-aminolevulinic acid ALA in vitro. Two groups were compared, one with ALA application and the other without at 19–37 degrees C and 37–46 degrees C. The level of cellular protoporphyrin IX (PpIX) was determined by high-performance liquid chromatography with fluorescence detection. Scanning laser confocal microscopy was used to observe the strength of fluorescence in Hep-2 cells. The ratio of cell death was detected using a flow cytometer before and after the photodynamic reaction. The authors’ pre-treatment results confirm that moderately higher temperature increases PpIX production and photodynamic response in ALA-induced human laryngeal squamous cell carcinoma in vitro [125].

Dima V.F. et al. evaluated Hep-2 human laryngeal cancer cells in their study. The cells were sensitized with different concentrations of hematoporphyrin and irradiated with a He-Ne laser of different fluence. They demonstrated a strong correlation between different PDT exposures and the degree of biochemical and ultrastructural changes in human laryngeal cancer cells in vitro [126].

Shafirstein et al., in their investigation to assess the safety of 3-(1′-hexyloxyethyl) pyropheophorbide-a (HPPH)-mediated PDT therapy for early-stage cancer, report that this therapy can be used safely in the early stages of the disease, with the restriction that the light dose does not exceed 100 J/cm^2^ at 4 mg/m^2^ HHPH and that patients are monitored for laryngeal edema immediately after therapy. The authors also conclude that first-stage cancerous lesions respond better to HPPH-PDT therapy than dysplasia (precancerous stage). Regarding side effects, Shafirstein et al., like Liang et al., mention laryngeal edema but also temporary hoarseness [127].

Yslas E.I. et al. investigated the use of ZnPcOCH3 PDT to treat the human laryngeal cancer cell line Hep-2 in vitro. They analyzed cellular uptake, lysosome changes, the process of death induced in Hep-2 cells, mitochondrial changes, and localization. The study showed the effectiveness of using ZnPcOCH3 as a potential photosensitizer in photodynamic therapy of human laryngeal cancer cell lines. Hep-2 cells show sensitivity to the phototoxic effects of ZnPcOCH3 [128].

Milanesio M.E. et al. compared the photodynamic effect of 5-[4-(trimethylammonium)phenyl]-10,15,20-tris(2,4,6-trimethoxyphenyl) porphyrin iodide with that of uncharged 5-(4-aminophenyl)-10,15,20-tris(2,4,6-trimethoxyphenyl)porphyrin. The authors used a homogeneous medium containing photo-oxidizable substrates, as well as in vitro human laryngeal cancer cell lines Hep-2. Comparing the results showed a higher phototoxic effect. Thus, a higher photo cytotoxic effect was found for uncharted 5-(4-aminophenyl)-10,15,20-tris(2,4,6-trimethoxyphenyl) porphyrin. This suggests the use of this substance as a potential phototherapeutic agent, suggesting its possible application in the inactivation of cancer cells through photodynamic therapy [129].

Milanesio M.E. et al., in their work, compared the photodynamic activity of porphyrin-C60 diadem (P-C60), and its zinc(II) metal complex (ZnP-C60) was investigated in comparison to 5-(4-acetamidophenyl)-10,15,20-tris(4-methoxyphenyl)porphyrin (P). They used a homogeneous medium containing photo-oxidizable substrates, as well as in vitro Hep-2 laryngeal cancer cell lines. The results showed that P-C60 has higher phototoxicity and was able to inactivate 80% of cells after 15 min of exposure. The authors also highlighted the retention of photoactivity by both diadems even in an argon atmosphere. Both compounds can cause biological photodamage through an O2(1 delta(g))-mediated photoreaction process or a free radical mechanism at low oxygen concentrations, depending on the microenvironment Through the ability of molecular diadems to create a photoinduced charge-separated state, they become a potential source of application in cell inactivation by PDT [130].

The potential of photodynamic therapy in the treatment of laryngeal cancer was also noted by Dailton Guedes de Oliveira Moraes et al. [20]. The authors, conducting in vitro studies, investigated the effect of PDT with the photosensitizer aluminum phthalocyanine tetrasulfonate (AlPcS4) on cancer cells. The authors showed positive results for damage to nuclear cells and the subcellular structure of carcinogenic cells using photodynamic therapy [131].

Degirmenci A. et al. show that the decoration of BODIPY with the 2,3-dihydrophthalazine-1,4-dione scaffold enhances singlet oxygen production. The treatment of Hep-2 epidermoid laryngeal carcinoma cells with the conjugates results in efficient cellular internalization, providing live imaging of Hep-2 cells and highlighting in vitro cytotoxicity after red light illumination [132].

Kleemann D. et al. examined the use of photodynamic therapy for treating laryngeal tumors with a focus on preserving normal tissue. Two photosensitizers, protoporphyrin IX (PPIX) induced by 5-aminolevulinic acid (ALA) and disulphonated aluminum phthalocyanine (AIS2Pc), were investigated. Peak PPIX levels were found in the mucosal epithelium, with necrosis limited to the mucosa at lower ALA doses and extending to muscle at higher doses. AIS2Pc primarily accumulated in the submucosa and muscle initially, moving to the mucosa over time, causing deeper necrosis at 1 h but limited to the mucosa at 24 h. Mucosal necrosis healed by regeneration, while deeper effects resulted in some fibrosis. Notably, no damage to cartilage was observed. Both photosensitizers are suitable for treating laryngeal mucosal lesions, but optimizing drug doses and the time interval between drug and light exposure is crucial to avoid undesirable changes in normal tissue [133].

Zhang, Chi et al. investigated the potential of photodynamic therapy to enhance vocal fold, enhance wound healing, and minimize scar formation, which are objectives in both preventative and restorative medical procedures on Sprague Dawley rats. Vocal fold stripping was performed, and PDT was administered using 5-Aminolevulinic Acid (5-ALA) and a 635 nm laser at different energy levels (20, 40, and 60 J/cm^2^). PDT was applied immediately post-surgery for prophylactic evaluation and four weeks after surgery for remodeling evaluation. Gene expression and histological assessments were conducted. PDT exhibited similar positive effects and contributed to vocal fold wound healing in both preventative and corrective procedures. It led to increased expressions of specific genes related to wound healing, including MMP8, MMP13, HAS2, and TGFβ1. Histological analysis revealed heightened vocal fold thickness, diminished collagen density, and increased deposition of hyaluronic acid in the lamina propria. Immunohistochemistry indicated improved collagen distribution and reduced density of collagen types I and III with the most pronounced changes observed in the 60 J/cm^2^ PDT group. The study concluded that PDT significantly enhances vocal wound healing, offering both prophylactic and remodeling effects. This minimally invasive treatment approach may be valuable for addressing vocal fold lesions with minimal scarring and could be useful in the treatment of acute or chronic vocal injuries to reduce scarring [134].

de Paula Rodrigues, Rafael et al. explored combining photodynamic therapy with quercetin (QCT) as a complementary approach in cancer treatment. QCT is known for its efficacy in reducing cell viability in various cancer cell lines. The research focused on evaluating different QCT concentrations in PDT’s impact on the viability, mitochondrial membrane potential, and induction of apoptosis/necrosis in human larynx carcinoma cells (HEp-2). HEp-2 cells were treated with aluminum phthalocyanine tetrasulfonate (AlPcS4) and QCT and then exposed to diode laser light (685 nm, 35 mW, 4.5 J/cm^2^). High concentrations of QCT (at least 50 μM) were found to reduce cell viability. This response was enhanced when combined with PDT. The study also observed a decrease in the mitochondrial membrane potential and characteristics of late apoptosis and/or early necrosis. QCT at concentrations of 50 μM or higher improved PDT-induced cytotoxicity by significantly reducing cell viability through apoptosis and/or necrosis while affecting mitochondrial membrane potential in HEp-2 cells [135].

Abramson, A. L. et al. employed a gold vapor laser emitting short 628 nm wavelength pulses to apply varying energy levels (ranging from 30 to 120 J/cm^2^) to canine larynx that had been sensitized with dihematoporphyrin ether. The results showed that at energy levels between 30 and 60 J/cm^2^, the larynx appeared normal. When the energy was increased to 70 to 100 J/cm^2^, only mild edema and erythema (redness) were observed. Simultaneously, a temperature increase of 0.4 °C to 1.5 °C was measured using a thermocouple probe. Consequently, the gold vapor laser was found to effectively achieve therapeutic photosensitization without causing significant laryngeal edema or notable thermal effects [136].

In a study by Sieron, A. et al., PDT was administered to two patient groups. Five patients with advanced tumors, including those with local recurrence (squamous cell carcinoma) following surgery and radiotherapy, received delta-aminolaevulinic acid (ALA) orally. Additionally, five patients with leukoplakia received an ointment containing 10% ALA applied locally. The tissue level of protoporphyrin IX was assessed before irradiation using specialized equipment. In the group with advanced tumors, a partial response was observed, leading to a reduction in cancerous ulcerations. In the leukoplakia group, a complete response was achieved in four out of five patients. These preliminary findings suggest that PDT may be beneficial for eliminating premalignant lesions in the oral cavity and providing palliative treatment for advanced lesions in the oropharynx and larynx [137].

Zhang, Chi et al.’s work consisted of ex vivo and in vivo experiments. In ex vivo tests on canine vocal folds, a 20% 5-ALA solution was applied topically. The depth of penetration and concentration of 5-ALA within the tissue were measured through various incubation times and application methods. In in vivo testing on leporine vocal folds, 5-ALA solution was sprayed once, twice, or administered systemically. The location and concentration of protoporphyrin IX (PPIX) were determined through fluorescence microscopy and a fluorescent quantitative method. Hematoxylin and eosin (H&E) staining was performed to visualize histological changes in the vocal folds after PDT. Experiments revealed that a 15 min topical incubation of 5-ALA achieved sufficient penetration depth (over 2 mm) and similar concentrations within the superficial 500 μm of the epithelium compared to longer incubation times. The topical spraying of 5-ALA produced adequate concentrations in vocal folds, although the retention time was brief. In an in vivo leporine model, laryngeal spraying of a 20% 5-ALA solution achieved similar penetration depth and PPIX concentrations as systemic 5-ALA administration. It was found that two sprays of a 20% 5-ALA solution with a 30 min interval were necessary for complete exfoliation of vocal fold epithelium. Topical PDT using laryngeal spraying of a 20% 5-ALA solution proved to be effective in achieving therapeutic results and holds potential for treating vocal fold leukoplakia [138].

von Beckerath, Mathias P. et al. considered a viable and safe treatment option for early-stage laryngeal cancer cases that may not be well-suited for radiation or trans-oral laser surgery (TLS). PDT demonstrates comparable cure rates to conventional therapies and yields voice outcomes on par with them. For certain sarcomas, PDT proves to be an organ- and function-preserving treatment, even though it tends to be more costly than other options. The study assessed PDT as the primary treatment and examined survival rates, tumor response, side effects, voice outcomes, and associated costs. The median follow-up period was 59 months. Out of ten patients, nine were successfully cured of their laryngeal cancer, with seven patients responding positively to PDT alone. All four sarcomas were effectively treated with temoporfin. Two out of three tumors involving the anterior commissure were cured using only interstitial illumination with PDT. Importantly, no severe side effects were observed. Furthermore, in 5 out of 10 cases, patients reported an improvement in their voices after treatment, and none experienced worsened voice quality [139].

Silbergleit, Alice K. et al. analyzed vocal fold vibration in patients with Tis-T1N0M0 squamous cell carcinoma (SqCCa) laryngeal tumors treated with photofrin-mediated photodynamic therapy (PDT). The hypothesis was that key vocal fold vibration attributes would return to baseline within 1–6 months of treatment. Laryngo Videostroboscopy data of eight patients were examined, with baseline and post-treatment vocal fold characteristics assessed by specialists in voice disorders. The study found that significant improvements in mucosal wave and amplitude of vibration occurred at 20 or more weeks post-PDT compared to the baseline. Comparing the results within 5 weeks post-procedure to 10–19 weeks post-procedure showed significant improvements in the amplitude of vibration and non-vibrating portions of the vocal fold. Comparing the results within 5 weeks post-procedure to 20 or more weeks post-procedure revealed significant improvements in the amplitude of vibration, mucosal wave, and non-vibrating portions of the affected fold. Photofrin-mediated PDT is effective in preserving laryngeal function and structure without systemic toxicity. However, it may take 4–5 months or more for key vocal fold vibration characteristics to recover after treatment [140].

Zhou, C. Y. et al. explored the therapeutic effect of photodynamic therapy (PDT) for the treatment of juvenile-onset laryngeal papillomatosis. Twenty-eight children with laryngeal papilloma were treated, with most having undergone more than four surgeries outside the hospital. The procedure involved visible tumor resection under a self-retaining laryngoscope and microscope, followed by the application of 20% 5-aminolevulinic acid (photosensitizer) and subsequent exposure to 635 nm semiconductor laser PDT with specific parameters. Among the 24 cases followed up for over a year (12 for 3 years), 19 showed no recurrence, resulting in a cure rate of 79.2%. Five cases experienced recurrence (20.8%), with three of them achieving a cure after three PDT sessions. The main complication was laryngeal adhesion. PDT with topical drugs for treating juvenile-onset laryngeal papillomatosis showed promising preliminary results. The paper also discussed the principles of PDT and factors contributing to the recurrence of laryngeal papilloma in children [141].

Peng, Zhongzhong et al. aimed to investigate the therapeutic effects of surgery, radiotherapy, and photodynamic therapy (PDT) on early glottic carcinoma (Tis-T2N0M0) and identify prognostic factors. A retrospective analysis was conducted on 202 cases of early glottic carcinoma treated with surgery (152 cases), radiotherapy (20 cases), and PDT (30 cases) between 2000 and 2013. Various parameters, including the Karnofsky performance status (KPS) score, disease-free survival (DFS), overall survival (OS), local control (LC), larynx preservation rate, and laryngeal function, were evaluated. Statistical tests and regression analyses were used for data analysis. There were no statistically significant differences in OS, DFS, and LC among the three treatment groups. The laryngeal function preservation rate was significantly higher in the radiotherapy and PDT groups (90% and 86.7%, respectively) compared to the surgery group (65.1%). However, there was no significant difference between the radiotherapy and PDT groups. Single-factor analysis revealed that the KPS score before treatment, vocal fold mobility limitation, and differentiation degree could impact prognosis. Multivariate regression analysis identified anterior commissure invasion, T stage, and KPS score before treatment as independent adverse prognostic factors for OS. T stage and differentiation degree were adverse prognostic factors for DFS, and T stage was also an adverse factor for LC. The occurrence of local recurrence or cervical lymph node metastasis did not significantly differ between the three groups. The therapeutic effects were similar in the surgery, radiotherapy, and PDT groups, with all three treatment modalities demonstrating good clinical results. Radiotherapy and PDT may be considered the primary or important treatments for early-stage glottic squamous cell cancer (Tis∼T2N0M0). However, the study’s limited sample size for PDT in T2 disease makes it challenging to draw definitive treatment conclusions for this subgroup [142].

Abramson, A. L. et al., in their work, showed the photodynamic activation of a hematoporphyrin derivative, originally designed for cancer treatment, and tested it as a potential therapy for papillomavirus infections of the larynx. In this study, two patients with the adult-onset form of this disease received the intravenous hematoporphyrin derivative (6 mg/kg) 72 h before endoscopic surgery. During surgery, 32 J/cm^2^ of light at 630 nm wavelength was delivered to the endolarynx using an argon pump dye laser. Notably, no significant complications, such as swelling or hemorrhage, were observed. A 13-month follow-up showed no recurrence of laryngeal papilloma in either patient. The study discusses the surgical technique, molecular biology, and clinical implications of using this approach to manage laryngeal papillomatosis [143].

Schweitzer, V. G. aimed to assess Cis-T2N0M0 squamous cell carcinoma (SqCCa) of the larynx in patients who were either unsuitable for or had not responded to traditional head and neck treatments. Over 15 years, ten patients with early-stage Tis-T2N0M0 SqCCa in the oral cavity and oropharynx, and another ten with Tis-T2N0M0 SqCCa in the larynx, were treated. The treatment involved administering intravenous PHOTOFRIN (porfimer sodium) at a dose of 2.0 mg/kg as an outpatient procedure. Subsequently, intraoperative light photoactivation at 630 nm was carried out using fiber-optic microlens (ML) delivery or cylindrical diffuser (CD) delivery, with specific light doses. In patients with diffuse field cancerization of the oral cavity, eight out of ten achieved complete responses (CRs) within a follow-up period of 6 months to 9 years. In individuals with superficial laryngeal cancer, eight out of ten also attained complete responses (CRs), thereby eliminating the necessity for total laryngectomy in patients who had undergone previous radiation therapy. PHOTOFRIN-mediated PDT provides a surgical oncologic treatment option for the potentially curative management of early-stage oral cavity and laryngeal malignancies. Notably, this approach is linked to minimal side effects, lacks systemic toxicity, preserves oral function and voice quality, permits multiple drug administrations, and allows for the possibility of laser light retreatment [144].

Ramon A. Franco Jr. conducted a study with the objective of assessing the safety and efficacy of aminolevulinic acid photodynamic therapy (ALA-PDT) in conjunction with the 585 nm pulsed dye laser for the treatment of laryngeal keratosis. Twelve male patients with keratosis were included in the study. A 20% ALA solution was applied as a spray into the larynx and subsequently activated using the 585 nm pulsed dye laser. Four patients were excluded for various reasons, leaving eight patients in the study. A total of 28 procedures were performed, with 64% of them taking place in a clinical setting. The study found a 78% reduction in keratosis (ranging from 10% to 100%). The study reported no significant or major side effects associated with the treatment. The mean follow-up period was 34.5 months, with a range of 12 to 50 months. No notable distinctions in outcomes were identified between treatments administered in an outpatient setting and those conducted in an operating room. The study concluded that ALA-PDL PDT demonstrated effectiveness and safety in treating laryngeal keratosis within an outpatient clinic environment. The procedure reduced morbidity without compromising treatment efficacy [145].

### 3.5. PDT Therapy in Oral Cavity

#### 3.5.1. HPPH-PDT

In two consecutive dose escalation investigations, Rigual N. et al. assessed the safety and efficacy of photodynamic therapy (PDT) using 3-(1′-hexyloxyethyl)pyropheophorbide-a (HPPH) for dysplasia and early squamous cell carcinoma in the head and neck. The studies involved 40 patients with oral dysplasia, carcinoma in situ, or early-stage head and neck squamous cell carcinoma (HNSCC). HPPH-PDT showed common adverse events of pain and treatment site edema. Complete response rates were 46% for dysplasia and carcinoma in situ and 82% for squamous cell carcinoma lesions at 140 J/cm^2^. However, responses in the dysplasia/carcinoma in situ cohort were not durable. The study emphasized the significance of STAT3 cross-linking as an indicator for assessing the photoreaction induced by HPPH-PDT [146]. McCaw D.L. et al. treated eleven dogs with oral squamous cell carcinomas using PDT with the photosensitizer Photochlor (HPPH). Photochlor was injected intravenously, and tumors were treated with 665 nm light. Eight dogs were considered cured, with no tumor recurrence for at least 17 months post-treatment. PDT demonstrated effectiveness comparable to surgical removal but with superior cosmetic results [147]. Rohrbach D.J. et al. investigated interlesion differences in PDT efficacy for two patients with oral lesions treated with the same drug dose and a similar light dose of HPPH-mediated PDT. The study utilized diffuse optical methods to quantify hemodynamic parameters and HPPH photosensitizer content, revealing substantial differences between lesions. PDT-induced changes, particularly the oxidative cross-linking of STAT3, exhibited significant variation between lesions [148]. In another study, Rohrbach D.J. et al. aimed to assess whether diffuse optical spectroscopy (DOS) measurements could effectively evaluate the clinical response to PDT in patients with early-stage head and neck squamous cell carcinoma. Thirteen patients received HPPH, and DOS measurements were conducted before and after PDT in the operating room. The change in HPPH concentration emerged as the most effective predictor of pathological response, and a two-parameter classifier achieved 100% sensitivity and specificity in classifying pathological response. DOS parameters could assess clinical response in the operating room, facilitating earlier reintervention if needed [149].

#### 3.5.2. AFL-PDT

Yao Y. et al. conducted a study comparing ablative fractional laser-assisted photodynamic therapy (AFL-PDT) to ablative fractional laser (AFL) treatment for oral leukoplakia (OL) in 48 randomized patients. The primary endpoints were efficacy and clinical recurrence, with side effects as a secondary endpoint. The AFL-PDT group showed a 100% effective cure rate, significantly higher than the AFL group (80.9%). Recurrence rates at 6 and 12 months post-treatment were lower in the AFL-PDT group. No severe adverse events or systemic side effects were reported in either group [150]. In a related study, Yao YL. et al. evaluated the results of AFL-PDT in treating OL and investigated risk factors associated with recurrence and malignant transformation. In 48 patients, AFL-PDT resulted in an overall positive response rate of 87.5%, with 62.5% achieving complete responses. Over a 3-year follow-up, recurrence and malignant transformation rates were 37.5% and 8.3%, respectively. Lesions on the gingiva/palate were associated with recurrence, and a higher risk of malignant transformation was linked to the severity of epithelial dysplasia and recurrence. AFL-PDT was effective for OL but required vigilant follow-up, with lesion location and epithelial dysplasia severity influencing outcomes [151].

#### 3.5.3. Oral Squamous Cell Carcinoma

Exploring various facets of photodynamic therapy (PDT) for early oral squamous cell carcinoma (OSCC), researchers have delved into innovative approaches and investigated the efficacy of different photosensitizers. Hopper C. et al. utilized meta-tetrahydroxyphenylchlorin (mTHPC) and reported a high efficacy, with an 85% complete tumor response in 114 protocol-compliant patients. The sustained complete response at 1 year (85%) and 2 years (77%) was coupled with favorable cosmetic and functional outcomes. The actuarial survival rates at 1 and 2 years were 89% and 75%, respectively, with manageable side effects, including mild-to-moderate pain and skin photosensitivity reaction [152].

Examining synthesized Pheophorbide a (Pa)-PDT in oral squamous cell carcinoma (OSCC) cells, Yoon H.E. et al. demonstrated a dose-dependent inhibition of cell proliferation, induction of apoptosis, and autophagy. In an in vivo murine model, intratumoral Pa-PDT inhibited tumor growth, emphasizing its potential therapeutic strategy for OS [153].

He X. et al. explored the combination of the epidermal growth factor receptor (EGFR) inhibitor nimotuzumab with aminolevulinic acid-induced photodynamic therapy (ALA-PDT) in OSCC. The combination therapy exhibited enhanced inhibition of OSCC cell growth in vitro and in vivo, leading to increased apoptosis and reduced EGFR expression, suggesting a promising approach for OSCC treatment [154].

Delving into the development of resistance to multiple cycles of PDT in OSCC cells, Rosin F.C.P. et al. revealed a survival phenotype characterized by reduced endogenous protoporphyrin IX (PpIX) expression, enhanced migration capacity, and the upregulation of pro-survival proteins. The study indicated that OSCC cells can develop resistance to PDT, raising the need for further exploration of the clinical implications of this resistance phenotype [155].

In a comprehensive review, various authors discussed light stimulus-responsive therapies, focusing on PDT, photothermal therapy (PTT), and light-triggered drug delivery systems in the treatment of OSCC. The article introduced various photosensitizers and nanomaterials, emphasizing their potential to enhance clinical outcomes for OSCC patients [156].

Examining the consequences of employing PDT with the photosensitizer 5-aminolevulinic acid (5-ALA) on OSCC, Pinto M.A.F. et al. revealed a notable reduction in cell viability and migration, affecting cancer stem cell (CSC) phenotypes and inducing differentiation [157].

Investigating prolonged outcomes of PDT on OSCC and epithelial dysplasia, Narahara S. et al. indicated good short-term outcomes but limited long-term effects. Surgical resection was performed for recurrences, suggesting the importance of alternative interventions [158].

Exploring the mechanism and effects of hematoporphyrin monomethyl ether-mediated PDT on OSCC, Meng P. et al. demonstrated inhibition of cell proliferation through the P53-miR-21-PDCD4 axis [159].

Examining the efficacy of combining PDT with the EGFR inhibitor nimotuzumab in OSCC, Bhuvaneswari R. et al. showed synergistic effects in delaying tumor growth, downregulating EGFR, and demonstrating no treatment-related toxicity [160].

Investigating topical ALA-PDT for early-stage lip SCC, Wang P. et al. achieved a complete response with no relapse during a 24-month follow-up for most patients, suggesting it as a potential alternative therapeutic option [161].

Exploring the therapeutic efficacy of PDT using the synthetic photosensitizer pheophorbide a (Pa-PDT) in AT-84 murine OSCC cells, Ahn M.Y. et al. demonstrated significant inhibition of cell growth, intracellular reactive oxygen species generation, and in vivo tumor growth inhibition [162].

Investigating the antineoplastic potential of PDT mediated by an aluminum-phthalocyanine chloride nanoemulsion (AlPc-NE) against an OSCC cell line in vitro, Cangussu L.M.B. et al. showed that PDT with AlPc-NE significantly reduced cell viability, migration, and altered cell proliferation and TP53 expression [163].

Studying the impact of PDT with synthesized pheophorbide a (Pa) on human OSCC cells, Ahn M.Y. et al. revealed its inhibitory effects on cell proliferation, induction of apoptosis, and autophagy. The suppression of autophagy enhanced PDT-induced necrosis [164].

Investigating the correlation between immunohistochemical markers and the recurrence of OSCC and oral epithelial dysplasia (OED) following PDT, Uehara M. et al. found that vascular endothelial growth factor (VEGF) expression was significantly lower in the recurrence group, suggesting its potential as a predictive marker for PDT effectiveness [165].

Conducting surface illumination mTHPC-PDT for T1/T2 N0 OSCC patients, Jerjes W. et al. reported favorable outcomes with a 15.8% recurrence rate and 84.2% 5-year survival, comparable to other interventions but with lower morbidity [166].

Evaluating the application of topical ALA-PDT synchronized with platinum-based induction chemotherapy (TPF) in locally advanced OSCC, Wang X. et al. achieved a comprehensive response rate of 90.9% and suggested its safety as an addition to induction chemotherapy followed by surgery [167].

Assessing the effectiveness of PDT with talaporfin sodium, a second-generation photosensitizer, on oral squamous cell carcinoma (SCC), Ikeda H. et al. found a high complete response rate, prompt elimination from the body, and efficacious treatment for oral squamous cell carcinoma, with talaporfin sodium demonstrating advantages in terms of prompt elimination from the body compared to porfimer sodium [168].

#### 3.5.4. TB/TBO PDT

In a randomized clinical trial exploring treatment options for erosive-atrophic oral lichen planus (OLP), Jajarm H.H. et al. compared the effectiveness of toluidine blue-mediated photodynamic therapy (TB-PDT) with local corticosteroids. The experimental group underwent TB-PDT with a 630-nm GaAlAs laser, while the control group used a mouthwash with dexamethasone and nystatin. Both groups exhibited a significant reduction in lesion sign scores after treatment (*p* = 0.021 for the experimental group, *p* = 0.002 for the control group), and the intensity of lesions significantly decreased in both groups (*p* = 0.005 for the experimental group, *p* = 0.001 for the control group). However, the control group showed a significantly greater improvement in lesion intensity reduction and pain (*p* = 0.001, *p* < 0.001, respectively), suggesting that while TB-PDT with laser was effective in managing OLP, the control group achieved superior results in terms of lesion intensity reduction and pain improvement [169].

For the management of oral lesions caused by Paracoccidioides brasiliensis (Pb), the fungus responsible for paracoccidioidomycosis (PCM), Dos Santos L.F.M. et al. investigated the application of photodynamic therapy (PDT) with toluidine blue (TBO). TBO-PDT resulted in a significant generation of hydroxyl radicals and hypochlorite, showcasing its potential for inactivating Pb colonies. In clinical cases, TBO-PDT led to an immediate relief of pain, improved mouth opening, and enhanced ability to chew and swallow. The study suggests that TBO-PDT is a safe, cost-effective, and promising therapy for managing the oral manifestations of PCM [170].

Garcia M.T.J. et al. delved into the optimization of photodynamic therapy (PDT) in oral cancer treatment by assessing the impact of Tween 80^®^ (TW) as a penetration enhancer on the mucosal retention of Toluidine Blue O (TBO). Mucoadhesive gels containing TBO with or without TW were prepared and evaluated for their physicochemical properties, in vitro release profiles, and in vitro mucosal retention. The results indicated that 4% chitosan-based gels containing 5% TW and 1% TBO possess suitable mucoadhesive and rheological properties for oral mucosa application. While TW reduced TBO release, it enhanced its retention in the mucosa, suggesting the potential for optimizing PDT responses in oral cancer treatment [171].

#### 3.5.5. ALA-PDT

In a comprehensive assessment, Han Y. et al. demonstrated the clinical efficacy of aminolevulinic acid (ALA)-mediated photodynamic therapy (PDT) in Chinese patients with oral leukoplakia, achieving a robust response rate of 86.2% [172]. Notably, complete remission was observed in 55.2%, and partial remission in 31.0%, indicating the effectiveness of topical ALA-PDT, especially in cases involving dysplasia. This aligns with the findings of Selvam NP. et al., who reported promising outcomes with 10% ALA-mediated PDT, highlighting complete and partial responses without recurrence. Their study proposed ALA-PDT as a promising alternative for oral leukoplakia treatment, leading to a satisfactory reduction in lesion size without side effects [173]. Additionally, Sieroń A. et al. investigated PDT for oral leukoplakia, achieving a complete response in 10 out of 12 patients, supporting the notion that PDT could serve as a viable alternative for addressing premalignant lesions in the oral cavity [174].

In a broader context, a literature review comparing photodynamic therapy (PDT) to surgical resection for early-stage squamous cell carcinoma (SCC) of the oral cavity, based on 24 studies, found no statistically significant difference in the complete response and locoregional control between the two modalities [119]. This suggests that PDT is equally effective compared to primary surgical resection, providing a valuable option for function-preserving treatment.

Wang X. et al. delved into the effects of ALA-PDT on a human oral precancerous cell line (DOK) and an oral squamous cell carcinoma cell line (CAL-27) [175]. The study revealed that ALA-PDT inhibited proliferation in both cell lines, with CAL-27 cells exhibiting higher rates of apoptosis and reactive oxygen species (ROS) levels compared to DOK cells. This highlights the potential of ALA-PDT in targeting both precancerous and cancer cells in vitro, although further clinical investigations are warranted to understand potential variations in susceptibilities.

Yamamoto M. et al. investigated the enhancement of protoporphyrin IX (PpIX) accumulation and PDT efficacy in an oral cancer cell line, revealing the influence of ABCG2 and FECH activities [176]. Their study suggested that the combination of these inhibitors enhances PDT efficacy, particularly in the presence of serum, offering a potential strategy to improve outcomes in challenging conditions.

#### 3.5.6. OLP-PDT

In a multifaceted investigation, Cosgarea R. et al. explored the clinical and immunological efficacy of photodynamic therapy (PDT) as an alternative treatment for oral lichen planus (OLP) [177]. The study involved twenty OLP patients who underwent PDT sessions over a 14-day period. The results revealed statistically significant improvements in clinical parameters and quality-of-life (QOL) measures. Notably, PDT induced a reduction in the relative number of CD4+ and CD8+ T cells in mucosal OLP lesions, decreased plasma levels of CXCL10, and diminished numbers of activated peripheral CD4+ CD137+ and CD8+ CD137+ T cells, as well as IL-17-secreting T cells. The study concluded that PDT represents a novel therapeutic option for OLP, demonstrating both local and systemic anti-inflammatory effects.

Saleh W. et al. conducted a study to compare the effectiveness of photodynamic therapy using methylene blue (MB) as a photosensitizer with topical corticosteroids in treating erosive oral lichen planus [178]. The research involved twenty patients divided into two groups, with one receiving MB-PDT and the other topical corticosteroids. Both groups exhibited significant improvement in subjective and objective scores, as well as lesion size, from baseline to the 4th week. MB-PDT demonstrated a higher degree of improvement compared to topical corticosteroids, suggesting that PDT could be an effective treatment for erosive OLP with favorable outcomes and minimal side effects.

In another comparative study, Zborowski J. et al. aimed to assess the effectiveness of photodynamic therapy in comparison to steroid therapy for oral lichen planus [179]. The 12-week split-mouth clinical trial included 30 patients with bilateral OLP. PDT, utilizing methylene blue as the photosensitizer, and triamcinolone as the steroid carrier, demonstrated high rates of complete remission. PDT showed 33.3% immediate remission and 54.2% after 3 months, while triamcinolone exhibited 22.2% and 62.9%, respectively. After 3 months, PDT achieved a 52.7% reduction in lesion area, surpassing the 41.7% reduction with triamcinolone. The findings suggest PDT as a promising treatment option, particularly in cases of contraindications or resistance to corticosteroids. These studies collectively highlight the potential of PDT as an effective and versatile therapeutic approach for managing different aspects of oral lichen planus, encompassing both clinical and immunological dimensions.

## 4. Clinical Use, Advances, and Limitations in the Use of Photodynamic Therapy

Photodynamic therapy has emerged as a promising and innovative approach in the field of medical treatment, particularly in the management of head and neck diseases [180]. One of the primary applications of photodynamic therapy in head and neck diseases is its role in the treatment of certain types of cancers, such as squamous cell carcinoma [181,182,183]. In addition to cancer treatment, photodynamic therapy has shown promise in managing infectious diseases affecting the head and neck [184]. PDT can effectively combat microbial infections, including bacterial, viral, and fungal pathogens [185]. The photosensitizing agents, when activated by light, produce reactive oxygen species that damage the cellular structures of pathogens, thereby suppressing the infection [186]. This has implications in the treatment of conditions like chronic sinusitis, oral infections, and human papillomavirus (HPV)-related lesions [187]. Furthermore, PDT has demonstrated efficacy in mitigating inflammatory conditions of the head and neck, such as recurrent respiratory papillomatosis (RRP) and periodontal diseases [188]. By modulating the inflammatory response and promoting tissue healing, PDT offers a non-invasive and targeted approach to managing these disorders. This is particularly valuable in cases where conventional treatments may be associated with significant morbidity or have limited effectiveness [189]. Despite the potential benefits of photodynamic therapy, further research is needed to refine treatment protocols, optimize the selection of photosensitizing agents, and explore its applicability across a broader spectrum of head and neck diseases [190]. Additionally, clinical trials are essential to establish the safety and long-term effectiveness of PDT in diverse patient populations [191]. The potential clinical application of photodynamic therapy in head and neck diseases is a promising avenue for improving treatment outcomes while minimizing side effects [192]. With ongoing advancements in technology and a growing body of evidence supporting its efficacy, PDT is poised to play a significant role in the multidisciplinary approach to managing various conditions in the intricate anatomy of the head and neck [193]. Photodynamic therapy significant advances have been made in refining PDT protocols, enhancing its efficacy, and expanding its scope, but the approach is not without its limitations. One of the remarkable advances in PDT is its ability to provide highly targeted treatment [194]. Through the selective accumulation of photosensitizing agents in diseased tissues, PDT minimizes damage to surrounding healthy cells, contributing to its efficacy in cancer treatment and other localized conditions [195]. PDT has transcended its initial applications and found utility across multiple medical disciplines. From dermatology to ophthalmology and beyond, the versatility of PDT has expanded, demonstrating its adaptability in addressing a diverse array of diseases and conditions [196]. Ongoing research has led to the development of more efficient and selective photosensitizing agents. Advances in molecular biology and nanotechnology have facilitated the design of agents with enhanced absorption properties and improved targeting, thereby optimizing the therapeutic effects of PDT [197]. The integration of PDT with other treatment modalities, such as chemotherapy and immunotherapy, represents a significant advancement [198]. Combination therapies have shown synergistic effects, enhancing the overall therapeutic outcomes and expanding the applicability of PDT in complex disease scenarios [199]. Advances in understanding cellular and molecular mechanisms have enabled researchers to refine PDT, focusing on intracellular targeting. This allows for more precise and effective destruction of pathological cells, improving the therapeutic potential of PDT [200]. One of the primary limitations of PDT is its restricted tissue penetration, particularly in deep-seated tumors. The effectiveness of PDT diminishes with increasing tissue depth, limiting its application to superficial or accessible lesions [201]. Patients undergoing PDT often experience photosensitivity, necessitating precautionary measures to avoid prolonged sun exposure [202]. Additionally, effectively delivering light to the targeted area, especially in internal organs, poses a challenge that researchers are actively addressing. While PDT is effective in many cases, its success can be contingent on factors such as tumor type and stage [203]. In some instances, the treatment may result in an incomplete tumor response, necessitating additional interventions for comprehensive disease management [204]. The cost of PDT, including photosensitizing agents, equipment, and specialized light sources, can be a limiting factor. This poses challenges in terms of accessibility and affordability, especially in regions with resource constraints [205]. Like many therapeutic approaches, PDT may encounter issues of resistance and disease recurrence. Understanding the underlying mechanisms and developing strategies to overcome resistance is an ongoing area of research. Photodynamic therapy has witnessed substantial advances, making it a valuable and evolving tool in the medical arsenal [206]. The targeted nature of treatment, multidisciplinary applications, improved photosensitizers, and the exploration of combination therapies contribute to the continued growth of PDT [207]. However, limitations such as tissue penetration challenges, photosensitivity, incomplete tumor response, cost implications, and the potential for resistance underscore the need for ongoing research and innovation in the field. By addressing these challenges, the medical community can harness the full potential of photodynamic therapy, further enhancing its efficacy and expanding its role in the comprehensive treatment of various diseases [208]. Table 2 summarizes clinical trials in head and neck diseases.

## 5. Ethical Considerations in the Use of Photodynamic Therapy

While PDT holds great potential to improve patient outcomes, its use raises several ethical considerations that must be carefully addressed [209]. In the context of PDT, obtaining informed consent is paramount. Patients must be fully informed about the nature of the procedure, potential risks, benefits, and alternative treatment options [210]. The unique photosensitizing agents and light exposure involved in PDT may present specific considerations that patients need to comprehend for meaningful consent. Ensuring a comprehensive understanding of the procedure fosters respect for autonomy and enables patients to make informed choices about their healthcare. Ethical practice demands that healthcare professionals prioritize the well-being of patients [211]. In PDT, the minimization of harm involves meticulous planning to limit collateral damage to healthy tissues. Clinicians must strike a delicate balance between achieving therapeutic efficacy and preserving the functional integrity of surrounding structures, thereby upholding the principle of non-maleficence [212]. The ethical distribution of healthcare resources is a fundamental consideration. PDT, while holding promise, may have associated costs that can create disparities in access. Striving for equitable access to PDT ensures that its benefits are not limited to specific socio-economic groups. Ethical healthcare practice calls for policies and initiatives that promote accessibility and affordability, addressing potential disparities in treatment availability [213]. Monitoring the long-term safety and outcomes of PDT is essential for ethical practice. Understanding the potential for delayed side effects or adverse events requires ongoing surveillance and reporting [214]. Clinicians have an ethical obligation to contribute to the body of knowledge regarding the safety profile of PDT, fostering transparency and accountability in the medical community. Ethical considerations extend to the realm of research and innovation in PDT [215]. Rigorous evaluations of new photosensitizing agents and treatment protocols are crucial to ensure safety and efficacy. Researchers must adhere to ethical guidelines, conducting trials with integrity, transparency, and a commitment to the well-being of study participants [216]. PDT involves the collection and storage of sensitive patient information. Maintaining strict confidentiality and safeguarding patient privacy are ethical imperatives. Robust data protection measures must be in place to prevent unauthorized access and ensure the integrity of patient information, respecting the principle of beneficence [217]. In harnessing the therapeutic potential of photodynamic therapy, healthcare practitioners, researchers, and policymakers must navigate a complex landscape of ethical considerations. Prioritizing informed consent, minimizing harm, promoting equitable access, monitoring long-term safety, conducting responsible research, and safeguarding patient privacy are integral to ethical practice in PDT [218]. By upholding these ethical principles, the medical community can maximize the benefits of PDT while ensuring the well-being, autonomy, and dignity of those undergoing this innovative therapeutic approach [211].

## 6. Conclusions

Photodynamic therapy (PDT) is an innovative and versatile treatment method used in various medical fields, including oncology. These studies collectively showcase the diverse applications of PDT in otolaryngology, ranging from cancer treatment and wound healing to managing complications and premalignant lesions. The findings underscore PDT’s potential as a valuable tool in preserving function and structure in the head area. Photodynamic therapy has the potential to prevent the recurrence of many conditions, which appeared in several cases described in the studies. Therefore, further studies should be continued on the effects of PDT, its safety, and possible side effects of this method in the treatment of head area diseases. Recent research has focused on photodynamic therapy as a potential solution. Several studies have explored the impact of PDT, demonstrating that PDT can be used safely. Additional investigations into PDT sensitizers like Norsquaraine 1 and delta-aminolevulinic acid showed promise, particularly in enhancing the production of reactive oxygen species and promoting the photodynamic response in laryngeal carcinoma cells. These findings indicate the potential of PDT as a valuable treatment approach for laryngeal diseases. The overall impact of photodynamic therapy on the treatment of human diseases has been significant and continues to evolve across various medical fields. Cancer treatment with PDT has demonstrated efficacy in treating various cancers, including skin, lung, esophageal, and head and neck cancers. Its targeted nature reduces damage to healthy tissues, minimizing side effects compared to traditional treatments like surgery and radiation. Infectious diseases show promise in combating microbial infections, including bacterial, viral, and fungal pathogens. Its ability to selectively target pathogens without inducing resistance makes it a valuable option for infectious disease management. It is widely employed in dermatology for conditions like actinic keratosis, acne, and certain skin cancers. Its cosmetic benefits, minimal scarring, and short recovery times contribute to its appeal in dermatological treatments. In the ophthalmology department, PDT is utilized in treating certain eye conditions, such as age-related macular degeneration (AMD) and certain types of glaucoma. Its non-invasiveness and ability to preserve surrounding tissues make it a valuable option for ocular therapies. In the neurology department, PDT is being explored for the treatment of brain tumors and neurological disorders. Its potential to target specific regions within the brain with minimal damage to healthy tissues is a promising avenue for neurological interventions. Photodynamic therapy has made a substantial impact on the treatment landscape of human diseases. Its targeted and minimally invasive nature, coupled with its adaptability across various medical disciplines, positions PDT as a valuable and evolving tool in the broader spectrum of therapeutic options available to clinicians. As research and technological advancements continue, the scope and impact of PDT are likely to expand, offering new avenues for improved patient outcomes in the realm of human health.

## Figures and Tables

**Table 1:** Comparison of PDT, radiological therapy, and surgery treatment.

	Photodynamic Therapy	Radiological Therapy	Surgical Therapy
Treatment mechanism	PDT relies on the interaction of photosensitizing agents, light, and oxygen to induce localized cytotoxic effects. It is a non-invasive and targeted therapy that selectively damages abnormal cells while sparing surrounding healthy tissues [73]	Radiological therapies, including external beam radiation and brachytherapy, use ionizing radiation to damage DNA within cells. This affects both cancerous and healthy cells, with the goal of inhibiting the growth of malignant tissues [74]	Surgical interventions involve the physical removal of abnormal tissue, offering immediate tumor debulking and potential cure, but may be associated with postoperative complications [75]
Selectivity and precision	High selectivity for diseased tissues due to the preferential accumulation of photosensitizers. Precise targeting minimizes damage to adjacent normal structures [76]	Targets both cancerous and normal tissues, leading to potential collateral damage and adverse effects [77]	Provides precision in localizing and removing abnormal tissue but may lead to collateral damage to nearby healthy structures [78]
Invasiveness	Generally non-invasive, with light delivery through optical fibers. Minimal impact on surrounding structures [79]	Non-invasive externally but may cause internal tissue damage. Brachytherapy involves the insertion of radioactive sources into or close to the tumor [80]	Invasive, involving incisions and tissue removal, with associated risks of bleeding, infection, and scarring [81]
Side effects and complications	Generally well tolerated, with localized erythema and edema as common side effects. Photosensitivity is transient [82]	Can cause acute and chronic side effects, including radiation dermatitis, mucositis, and damage to surrounding structures [83]	Potential for complications such as bleeding, infection, and nerve damage. Postoperative recovery may be prolonged [84]
Repeatability	Can be repeated without cumulative toxicity, allowing for multiple treatment sessions [85]	Limited by cumulative radiation toxicity, with a maximum dose constraint [86]	Repeat surgeries may be challenging due to tissue scarring and patient recovery considerations [87]
Cost and accessibility	Equipment and photosensitizing agents may contribute to costs, but accessibility is generally good [88]	Equipment costs are high, and access may be limited in certain regions [89]	Associated with significant costs, including surgical facilities, anesthesia, and postoperative care [90]

**Table 2:** Summary of clinical studies (provided name of the photosensitizer, dose administered, and research group).

References	PS	PS Dose	Research Group
[91]	Verteprofin	3 mg/m^2^	179 (67 final half dose PDT)
[92]	Verteprofin	3 mg/m^2^	42 (32 half dose PDT)
[93]	Verteprofin	3 mg/m^2^	90 (42 half dose PDT)
[94]	Verteprofin	2 mg/m^2^ or 3 mg/m^2^	27
[95]	Verteprofin	6 mg/m^2^	12
[96]	Verteprofin	2 mg/m^2^	60
[99]	Verteprofin	6 mg/m^2^	322
[100]	Verteprofin	6 mg/m^2^	322
[101]	Verteprofin	6 mg/m^2^	1
[102]	Verteprofin	-	56
[103]	Verteprofin	6 mg/m^2^	48
[105]	BCECF-AM (2,7,-bis-(2-carboxyethyl)-5-(and-6)-carboxy-fluorescein, acetoxymethyl-ester)	80 μg/300 μL BSS	36
[106]	Verteprofin	6 mg/m^2^	9
[107]	Verteprofin	6 mg/m^2^	41
[108]	5-ALA	20% 5-ALA solution was applied on the tumor and its surrounding area of 0.5 cm of normal skin	1
[112]	Photofrin	2 mg/kg	30
[113]	mtetrahydroxyphenylchlorin	0.15 mg/kg	15
[114]	methylene blue	-	75 (37 PDT)
[116]	meta-tetraHydroxyPhenylChlorin (mTHPC or Foscan	0.15 mg/kg	11
[117]	5-ALA	0.4 cc, 20%	1
[118]	hematoporphyrin	-	13
[122]	5-ALA	118 mg/bottle	3
[123]	5-ALA	20% concentration solution	13
[127]	3-(1′-hexyloxyethyl) pyropheophorbide-a	4 mg/m^2^	29
[137]	delta-aminolaevulinic acid	3 g and ointment containing 10% ALA	10
[139]	temoporfin	0.15 mg/kg	10
	HpD	2 mg/kg 5 mg/kg	
[140]	photofrin	-	8
[141]	5-ALA	20% solution	28
[142]	photofrin	2 mg/kg	202 (30)
[143]	hematoporphyrin derivative	6 mg/kg	72
[144]	porfimer sodium	2.0 mg/kg	20
[145]	ALA	1.5 to 3.0 cc of 20% ALA was aerosolized and sprayed into the larynx	12
[146]	3-(1′-hexyloxyethyl)pyropheophorbide-a	4 mg/m^2^	40
[148]	2-1[hexyloxyethyl]-2-devinylpyropheophorbide-a	4.0 mg/m^2^	2
[149]	2-[1-hexyloxyethyl]-2-devinylpyropheophorbide-a	4.0 mg/m^2^	13
[150]	5-ALA	20% 5-ALA	48
[151]	5-ALA	20% 5-ALA gel	48
[152]	meta-tetrahydroxyphenylchlorin	0.15 mg/kg	121
[158]	porfimer sodium or talaporfin sodium	2 mg/kg	23
[161]	5-ALA	20% 5-ALA	6
[166]	meta-tetrahydroxyphenylchlorin	0.15 mg/kg	28
[167]	5-ALA	10% 5-ALA cream	11
[168]	talaporfin sodium	40 mg/kg	8
[169]	toluidine blue	50 μL toluidine blue (1 mg/mL)	25
[170]	hydroxyphenyl fluorescein and aminophenyl fluorescein	37.5 mg/L	3
[172]	5-ALA	20% 5-ALA gel	29
[173]	5-ALA	10% solution	5
[174]	delta-aminolevulinic acid	10% solution	12
[178]	methylene blue	5%	20
[179]	methylene blue	5%	30
[182]	5,10,15,20-tetra(m-hydroxyphenyl)chlorin (mTHPC)	0.15 mg/kg	128
[183]	temoporfin	0.15 mg/kg	39

- The concentration of the photosensitizer was not provided for the selected references.
